# First language acquisition differs from second language acquisition in prelingually deaf signers: Evidence from sensitivity to grammaticality judgement in British Sign Language

**DOI:** 10.1016/j.cognition.2012.04.003

**Published:** 2012-07

**Authors:** Kearsy Cormier, Adam Schembri, David Vinson, Eleni Orfanidou

**Affiliations:** Deafness, Cognition & Language Research Centre, University College London, 49 Gordon Square, London WC1H 0PD, England, United Kingdom

**Keywords:** Deaf, British Sign Language, Critical period, Acquisition, Grammaticality judgement, Syntax

## Abstract

Age of acquisition (AoA) effects have been used to support the notion of a critical period for first language acquisition. In this study, we examine AoA effects in deaf British Sign Language (BSL) users via a grammaticality judgment task. When English reading performance and nonverbal IQ are factored out, results show that accuracy of grammaticality judgement decreases as AoA increases, until around age 8, thus showing the unique effect of AoA on grammatical judgement in early learners. No such effects were found in those who acquired BSL after age 8. These late learners appear to have first language proficiency in English instead, which may have been used to scaffold learning of BSL as a second language later in life.

## Highlights

► Deaf native signers, early and late learners judged BSL sentence grammaticality. ► Early learners performed worse the later they were exposed to BSL. ► Late learners’ performance was not affected by age of learning BSL. ► Unique effect of age of learning BSL found in early learners. ► Prelingually deaf late learners may benefit from first language competence in English.

## 1 Introduction

According to a hypothesis originally proposed by Lenneberg (1967), there is a critical (or sensitive) period for acquisition of a first language (L1) – i.e., the first accessible language to which an individual is exposed, typically from birth – linked to neural plasticity which decreases as an individual grows older. This hypothesis has also been extended to apply to subsequent, second language (L2) acquisition. Based on a variety of studies which have examined L2 acquisition (e.g., Birdsong, 1999b), it does indeed seem clear that earlier exposure to a second language leads to more native-like competence of that language, although whether an actual critical period exists for L2 acquisition due to changes in the brain is unclear. Some have argued for a critical period for L2 acquisition, based on studies showing that native-like proficiency declines when the age of acquisition is after puberty (e.g., Coppieters, 1987; Eubank & Gregg, 1999; Johnson & Newport, 1989; Weber-Fox & Neville, 1999). However, others have argued against a critical period based on studies showing that individuals acquiring a second language may achieve near-native levels of proficiency at any age (e.g., Birdsong, 1992; Bongaerts, 1999; Hakuta, Bialystok, & Wiley, 2003; White & Genesee, 1996).^3^

Unlike the L2 critical period hypothesis, the L1 critical period hypothesis is untestable in the general population, because all typically-developing, hearing children have access to a first language from the surrounding language community. Evidence to support the L1 critical period hypothesis comes primarily from two groups with atypical language development. One group is children who have been deprived of language via isolation from humans early in life. One well-known case is Genie, a child who was isolated for the first 13 years of her life. After she was discovered and exposed to English at age 13, Genie exhibited better lexical than syntactic development and better comprehension than production. Her rate of language acquisition was overall very slow as well, and she had long-lasting problems with particular grammatical structures. However, it is difficult to draw strong conclusions about language development from cases such as Genie, not only due to their extreme rarity, but also because their isolation has broad consequences on all aspects of cognitive and social development, not only their linguistic development; thus any effects of age of language acquisition are confounded with these other variables (Curtiss, 1977; Skuse, 1988).

The second group which provides evidence for a critical period for L1 acquisition are profoundly deaf children born into hearing families. In North America, for example, language acquisition and transmission for deaf individuals differs substantially from that of hearing individuals, with a maximum of only 5–10% of deaf children acquiring a sign language natively from deaf or hearing signing parents (Mitchell & Karchmer, 2004). This minority of deaf children who are exposed to a sign language from birth acquire that sign language in much the same way that hearing children acquire their native spoken language and with similar maturational milestones (Newport & Meier, 1985; Petitto & Marantette, 1991). The remaining 90–95% of deaf children are born to hearing families who typically do not use a sign language, instead focusing on teaching the child spoken language. Such an approach has had typically highly variable outcomes. Spoken language acquisition, if it does occur, is significantly delayed in many deaf children compared to hearing children, depending not only on the degree and kind of hearing loss but also the child’s home and school environments, their intelligence and time spent reading (Blamey, 2003; Sarant, Holt, Dowell, Rickards, & Blamey, 2009). In terms of the school environment, there have traditionally been three main types of communication that schools use in the education of deaf students: auditory-oral methods (where the focus is on speech, listening and/or speechreading), bimodal methods (where artificial sign systems are used to represent spoken language, e.g., Signed English or Sign Supported English in the UK), and natural sign languages such as British Sign Language (BSL). During the twentieth century, oral and bimodal methods were most prevalent worldwide until the 1980s which saw the introduction of bilingual–bicultural approaches to deaf education in some countries, due to changes in awareness of and attitudes towards the language and culture of deaf communities and the recognition that deaf children are equally as capable of learning as their hearing peers (Swanwick & Gregory, 2007). The basic premise of bilingual–bicultural education was that full access to education via a natural sign language would facilitate age-appropriate language and cognition; this would then provide the basis for a transition to text-based literacy in the majority spoken language (Cummins, 1989; Johnson, Liddell, & Erting, 1989; Lane, Hoffmeister, & Bahan, 1996). Bilingual–cultural models of deaf education are still popular today. However, a lack of empirical evidence to support the effectiveness of bilingual approaches, along with technological advances such as newborn hearing screening and cochlear implants, both pose possible challenges to the continuation of bilingual programmes for deaf children in the UK and worldwide (Mayer & Leigh, 2010; Powers, 2002).

Whether or not spoken language proficiency is successfully attained, some degree of proficiency in the surrounding spoken language via reading and writing can be achieved by some deaf individuals. However, such successes with literacy are again highly variable and not common. The average reading age for the adult deaf population in the UK and the USA is generally believed to be around 8 or 9 years, based on data from Conrad (1979) and Traxler (2000).

Regardless of their success or failure to acquire spoken or written language, many deaf individuals from non-signing families may begin to use a sign language. This may occur only later in childhood when they encounter signing in school, or even much later after having left school. Some of these individuals can be considered to have delayed acquisition of a sign language as L1 (i.e., those for whom acquisition of the surrounding spoken language as an L1 has clearly failed). However, many deaf individuals have some degree of proficiency in the surrounding spoken language, so determining true cases of delayed L1 acquisition of a sign language, as opposed to second language (L2) acquisition of a sign language after successful acquisition of the surrounding spoken/written language as L1, can be a challenge. Distinguishing these cases of late L1 versus L2 sign language acquisition is crucial for making claims about the L1 critical period hypothesis, since it is widely believed that first language acquisition differs from second language(s) in that the former typically leads to native proficiency. Although “the earlier, the better” applies in both cases, early acquisition is considered relatively less important for ultimate attainment of L2 skills compared to L1. Also, some research suggests that second language acquisition is not a good test for the notion of a critical period for language in any case, because the outcome of second language acquisition may be influenced by circumstances of first language acquisition (Werker & Lalonde, 1988). Therefore, first language acquisition is a better test of the notion of critical periods overall. Acquisition of a first language from birth will ultimately lead to native proficiency, whereas delayed first language acquisition is unlikely to lead to complete acquisition at all, much less native/near-native proficiency. However, as noted above, cases of delayed first language acquisition of a spoken language are rare and confounded with other variables. Deaf individuals acquiring a signed language at varying ages provide a better testing ground for hypotheses about L1 critical periods.

The current study examines the effects of age of acquisition on sensitivity to grammatical judgement in BSL. BSL is a natural sign language used by the deaf community in the United Kingdom (Sutton-Spence & Woll, 1999). Many aspects of its linguistic structure at the levels of phonology, morphology and syntax are different from English. BSL is also unrelated to, and for the most part mutually unintelligible with, most other sign languages, including American Sign Language (ASL) and Irish Sign Language (ISL).

Many previous studies have shown age of acquisition effects within sign languages such as ASL and BSL, at phonological, morphological, and syntactic levels. Mayberry and colleagues (Mayberry & Eichen, 1991; Mayberry & Fischer, 1989) found that native signers outperformed non-native signers of ASL in sentence recall and sentence shadowing tasks, while Emmorey and colleagues (Emmorey, 1991; Emmorey, Bellugi, Friederici, & Horn, 1995; Emmorey, Corina, & Bellugi, 1995) found age of acquisition effects in ASL morphological repetition priming, lexical decision, sign monitoring, and probe recognition tasks. Differences have also been found between native and non-native signers in brain activation. MacSweeney, Waters, Brammer, Woll, and Goswami (2008) reported greater activation in the left inferior frontal gyrus in deaf non-native signers than native signers during a BSL phonological judgement task, while Newman, Bavalier, Corina, Jezzard, and Neville (2002) found more right hemisphere angular gyrus activation in hearing, native signers of ASL (i.e., ASL-English bilinguals) than in hearing late learners of ASL. These data have been used in support of the L1 critical period hypothesis. However, some of these studies did not distinguish between first language and second language acquisition, because they did not control for the extent to which the non-native signers were proficient in a spoken language before acquisition of a sign language, an important issue when considering whether their sign language was an L1 or an L2. This is a crucial issue when considering whether the results are relevant to critical periods for L1 acquisition.

Some other studies have recognised the need to distinguish between L1 and L2 acquisition by attempting to ensure that their non-native signer participants had a sign language, rather than a spoken/written language, as L1. For example, Newport (1990) reported that native signers outscored non-native signers in a variety of production and comprehension tests of ASL morphology and syntax.^4^ Morford, Grieve-Smith, MacFarlane, Staley, and Waters (2008) found age of acquisition effects in perception of handshape and place of articulation in lexical signs of ASL. Specifically, deaf native signers (with exposure to ASL from birth) showed higher discrimination with handshapes that were on the periphery of the category prototype or which straddled the category boundary, compared to deaf late learners of ASL (age of acquisition ranging from 10 to 18 years old). Mayberry (1993) directly compared L1 acquisition with L2 sign language acquisition effects, by comparing sentence recall in deaf ASL signers who reported acquiring ASL as a first language at various ages – from birth, early (between ages 5 and 8), and late (between ages 9 and 13) – with postlingually deaf individuals who had acquired English as L1 and acquired ASL as L2 after becoming deaf later in childhood. In this study, Mayberry found age of learning L1 effects: native signers performed better than early learners who performed better than late learners. Those who had acquired ASL as an L2 performed more similarly to the early L1 learners than the late L1 signers. From this, Mayberry concluded that L1 acquisition of a sign language (in prelingually deaf individuals) differs from L2 acquisition of a sign language (in postlingually deaf individuals). Mayberry, Lock and colleagues (2003, 2002) extended these findings by examining L2 English proficiency in deaf L1 signers of ASL (with varying ages of acquisition) compared with L2 English proficiency in native speakers of spoken French, German, Italian and Greek. Their findings indicated that deaf native/near-native ASL signers (who had acquired English between ages 3 and 7 as L2) and hearing non-signers (who had acquired English between ages 6 and 13 as L2) performed better on English tests than deaf ASL signers who had acquired English between ages 6 and 13 as essentially a delayed first language.^5^ Finally, Boudreault and Mayberry (2006) examined grammatical judgement in signers who had acquired ASL as a first language from birth, early childhood, or later in life, finding that as delay in age of exposure increased, performance in grammatical judgement decreased, similar to Mayberry (1993).

Taken together, these studies on deaf populations strongly suggest that age of first language acquisition of a sign language affects ultimate proficiency in that language. However, in all of these studies, the status of ASL or BSL as the first language of the non-native signers was based on their inability to use spoken English (Boudreault & Mayberry, 2006; Mayberry, 1993; Mayberry & Lock, 2003) or their “limited knowledge of English” (Morford et al., 2008, p. 43) as determined by self-report, or their “limited skills in English” with no indication of how this was determined (Newport, 1990, p. 14). The problem with relying solely on self-report for determining L1 in deaf signers is that deaf individuals are typically bilingual to some degree (even if only a small degree) in the surrounding spoken and/or written language (Ann, 2001; Grosjean, 1992), and there is great heterogeneity in age of both L1 and L2 acquisition, as discussed above. Determining the degree of competence in spoken/written language via only self-report can be difficult, particularly in late learners who would have had more time to potentially develop proficiency in the spoken/written language. In most of these studies (MacSweeney et al., 2008, being one exception), measures which could have more convincingly eliminated the possibility of English as L1, such as reading ability (either via formal assessment or self-report), were not reported. In the current study, we examine age of L1 acquisition effects in a sign language by employing a grammaticality judgment task based on the ASL task designed by Boudreault and Mayberry (2006). Crucially, we take into account English reading performance and nonverbal IQ, in addition to self-reported demographic information from participants, in order to more directly assess the critical period hypothesis. Because our study is based closely on materials developed in ASL by Boudreault and Mayberry (2006), we first outline their methods and results.

## 2 ASL Grammaticality Judgement Task (Boudreault & Mayberry, 2006)

Boudreault and Mayberry (2006) designed an ASL grammaticality judgement task to test whether age of acquisition had differential effects with regard to off-line grammaticality judgement of different syntactic structures. They selected various morphosyntactic structures based on their reported developmental timecourse in ASL acquisition. This included ASL structures which have been claimed to be acquired relatively early (by age 2;6–3;0), such as basic word order with plain verbs (Pichler, 2002) and negation and ‘agreement’ verbs^6^ (Anderson & Reilly, 1997; Meier, 1987). Boudreault and Mayberry also included structures reportedly acquired later such as wh-questions, relative clauses, and ‘classifier’ constructions.^7^ Although elements of wh-questions and classifier constructions first occur at ages similar to simple structures (Anderson & Reilly, 2002), adult-like use of these structures in ASL has been found to follow a protracted timecourse with full mastery occurring between 4 and 9 years old (Lillo-Martin, 2000; Supalla, 1982). Boudreault and Mayberry conjectured that, although the acquisition of relative clauses has not been studied in ASL in any depth, the structural similarity between relative clauses and conditional structures (acquired by around 8 years of age, cf. Reilly, McIntyre, & Bellugi, 1990) suggests that relative clauses are also likely to be acquired late.

The stimulus set consisted of 14 pairs of grammatical and ungrammatical sentences based on each of six syntactic structures: simple sentences, sentences with negation, sentences with agreement verbs, questions, relative clause structures and classifier constructions. All ungrammatical stimulus sentences were created from the grammatical stimulus sentences by moving a constituent to an incorrect position in the sentence. Participants were instructed to focus on detecting errors in the structure of the stimuli, including non-manual marking (e.g., changes in facial expression), sign order and/or the use of space.

The participants in Boudreault and Mayberry’s study were 10 native signers of ASL (deaf signers from deaf families), 10 early learners of ASL (reported age of acquisition between 5 and 7 years of age) and 10 late learners of ASL (reported age of acquisition between 8 and 13 years of age). All participants used ASL as their preferred language and had been using ASL for at least 13 years. Further demographic information from the ASL participants, such as mean and range of ages of acquisition (AoA) and mean and range of ages and length of ASL use, are shown in Table 1. Importantly, Boudreault and Mayberry claimed that all of their participants were L1, not L2 learners of ASL: “No participant reported successful acquisition of a spoken language prior to learning ASL and none reported the ability to navigate everyday life through the exclusive use of speech and speech-reading” (p. 613). This latter criterion, determined by self-report only, was crucial for establishing that all of their participants acquired ASL *as a first language*.

Boudreault and Mayberry’s (2006) results showed large effects of age of acquisition on response accuracy (as shown in Table 1), but not response latency. Age of acquisition effects did not interact with the effects of morphosyntactic structure, although negative structures were most accurate overall and relative clauses the least accurate overall. With response accuracy, Boudreault and Mayberry found an interaction between grammatical status (i.e., grammatical versus ungrammatical) and AoA and also between grammatical status and syntactic structure. That is, participants made more errors with ungrammatical than grammatical stimuli across all structures except negative and classifier constructions, and this was particularly pronounced in the early and late learners. Thus, Boudreault and Mayberry found strong age of acquisition effects on accuracy, and also an interaction between grammaticality and AoA for most structures. They concluded from this that delayed L1 acquisition affects the ultimate acquisition of morphosyntax in ASL. Combined with Mayberry’s previous work (Mayberry & Eichen, 1991; Mayberry & Fischer, 1989), they argued that their results showed that delayed L1 acquisition affects all subsequent language acquisition.

## 3 BSL Grammaticality Judgement Task (BSLGJT)

The aims of the current study were twofold: (a) to test Boudreault and Mayberry’s (2006) claims made about delayed L1 acquisition of sign language morphosyntax using a BSL version of their ASL Grammaticality Judgement Task, thus exploring this claim for the first time in a behavioural study with a sign language other than ASL, and (b) to develop the BSL Grammaticality Judgement Task into a screening tool for assessing BSL skills in deaf adults, since no such tools existed when this study commenced. In addition to these two main aims, we also wished to rule out other possible explanations for Boudreault and Mayberry’s findings for ASL. We therefore included measures of nonverbal IQ and reading, which we regarded a proxy measure of English language proficiency. By factoring out reading proficiency and also nonverbal IQ, we argue we are more able to directly assess the effects of *first* language acquisition. Unlike Boudreault and Mayberry (as we explain in more detail later), we opted to include some participants who appeared to have L1 proficiency in English in our late learner group, as deaf individuals reporting that they had acquired BSL as an L1 during or after late childhood were difficult to recruit for this study. We assumed that our reading measures would correctly identify those signers with higher levels of English proficiency.

The six constructions included in the task by Boudreault and Mayberry – simple sentences, sentences with negation, sentences with agreement verbs, questions, relative clause structures and classifier constructions – have also been identified in many other sign languages (Cecchetto, Geraci, & Zucchi, 2006; Mathur & Rathmann, 2010; Pfau & Steinbach, 2005; Zeshan, 2006; Zwitserlood, in press), including BSL (Sutton-Spence & Woll, 1999). There are some ways in which these BSL syntactic constructions differ from those in ASL. One example is negation. ASL has a general negative adverbial sign not which is used in addition to a range of negative manual signs including signs such as never, nothing, not-yet, negative modals (can’t, won’t) etc., in addition to non-manual marking (i.e., headshake) (Wood, 1999). In BSL, observation suggests that negation is more commonly expressed either only non-manually (i.e., with a headshake) or via other negative manual signs such as never, nothing, not-yet, can’t, won’t, etc., rather than by a general manual negative adverbial sign not.^8^ Marking of relative clauses in ASL and BSL also differs. ASL uses the manual signs that and self as relative pronouns along with non-manual and prosodic marking. BSL uses similar non-manual and prosodic cues to mark relative clauses (Cormier, in press). However, to our knowledge, BSL does not have relative pronouns like ASL. Aside from these differences, these structures function essentially the same way in BSL and ASL.^9^ Therefore, in order to try to replicate the age of acquisition effects that Boudreault and Mayberry (2006) had found with these types of sentences in ASL, we used these same sentence types for BSL.

In terms of children’s development, the acquisition of syntactic structures in BSL has not been studied to the same extent as the acquisition of ASL syntax. It does seem clear that deaf children acquiring BSL are able to master verb agreement and classifier constructions around the same ages as deaf children acquiring ASL (Morgan, Barriere, & Woll, 2006), so it is not unreasonable to assume the timecourses for the other constructions are similar for BSL as well (i.e., earlier acquisition of basic word order in simple sentences, negation and agreement structures compared to later acquisition of questions, classifier constructions and relative clause structures).

## 4 Method

### 4.1 BSL stimuli

An initial set of 168 BSL stimuli were created by the second author of this paper (a fluent hearing signer and linguist) in consultation with deaf native signers, modelled on the ASL stimuli from Boudreault and Mayberry (2006). The stimulus set contained 14 pairs of grammatical and ungrammatical sentences based on six syntactic structures: simple sentences, and sentences with negation, agreement verbs, wh-questions, relative clauses and classifier constructions. The ungrammatical sentences were created by moving a constituent to an incorrect position in the sentence. All stimulus clauses were 6–9 morphemes in length. (Meaningful uses of non-manual marking and of spatial locations associated with referents were counted as ‘morphemes’ in the BSL study).

#### 4.1.1 Simple sentences

Fourteen pairs of simple sentences all contained plain, uninflected verbs, as in (1a). No agreement verbs or classifier constructions were used in these sentences. The ungrammatical version of each grammatical sentence was produced by moving the verb into an incorrect position in the subject noun phrase, as in (1b).

(1a) Simple sentence (grammatical)^10^
fifty year ago most man smoke
‘Fifty years ago, most men were smokers.’ (Supplementary Video 1)
(1b) Simple sentence (ungrammatical)
^*^fifty smoke year ago most man
‘Fifty smoke years ago, most men.’ (Supplementary Video 2)

#### 4.1.2 Negated sentences

Fourteen pairs of negated sentences contained plain, uninflected verbs. No agreement verbs or classifier constructions were used in these sentences. Seven of these pairs had only non-manual marking of negation (e.g., headshake), and the ungrammatical sentence was produced by moving the non-manual marking from the verb phrase to the subject noun phrase, as in (2a). The other seven pairs used a manual negator sign (a negative form of a modal such as can’t or won’t), along with the non-manual marker of negation, as in (3a). The ungrammatical sentences were produced by moving the modal and the non-manual marking to an incorrect position in the object noun phrase, as in (2b) and (3b).

(2a) Negative sentence with non-manual marking only (grammatical)
________________hs
poss-3 sister steal poss-2 camera
‘Her/his sister didn’t steal your camera.’ (Supplementary Video 3)
(2b) Negative sentence with non-manual marking only (ungrammatical)
__________hs
^*^poss-3 sister steal poss-2 camera
‘Not her/his sister steal your camera.’ (Supplementary Video 4)
(3a) Negative sentence with negative modal and non-manual marking (grammatical)
________________hs
pro-1 can’t fix poss-2 car
‘I can’t fix your car.’ (Supplementary Video 5)
(3b) Negative sentence with negative modal and non-manual marking (ungrammatical)
_______hs
^*^pro-1 fix poss-2 can’t car
‘I fix your can’t car.’ (Supplementary Video 6)

#### 4.1.3 Agreement verb sentences

Fourteen pairs of sentences contained an agreement verb as in (4a), with the ungrammatical sentence produced by moving the verb to an incorrect position in the object or subject noun phrase as in (4b). These sentences included sentences with double agreement verbs (in which the verb sign moves from a location associated with the subject argument to one associated with the object) and sentences with single agreement verbs (in which the verb sign moves from the body and/or a neutral location to a location associated with the object argument). No classifier constructions were used in these sentences.

(4a) Agreement verb sentence (grammatical)
________br
sign class poss-1 student++ reject-3
‘My students couldn’t be bothered with the sign class.’ (Supplementary Video 7)
(4b) Agreement verb sentence (ungrammatical)
__br
^*^sign reject-3 class poss-1 student++
‘Sign reject class my students.’ (Supplementary Video 8)

#### 4.1.4 Wh-questions

Fourteen pairs of sentences contained a wh-question sign, with seven pairs involving the use of plain verb as in (5a) and seven involving an agreement verb as in (6a). No classifier constructions were used in these sentences. The ungrammatical sentences were produced by moving the question sign to an incorrect position in the object or subject noun phrase, as in (5b) and (6b). We opted to use only clauses including wh-question signs for this section, whereas the original ASL task included both wh- and yes–no interrogative constructions (the latter were erroneously referred to as wh-questions in Boudreault & Mayberry, 2006). The wh-questions were marked with a wh-question sign and the associated non-manual marking (e.g., furrowed brows). The yes–no questions had only non-manual marking associated with yes–no questions (e.g., raised brows). In the ASL task, the non-manually marked yes–no questions were made ungrammatical by changing the scope of the required non-manual element, but as the syntactic versus prosodic nature of these non-manual features is in dispute in the literature (e.g., Sandler & Lillo-Martin, 2006), we decided not to include sentences of this type.

(5a) Example: Wh-question with plain verb (grammatical)
___________br _________bf
new house key who forget
‘Who forgot the key to the new house?’ (Supplementary Video 9)
(5b) Example: Wh-question with plain verb (ungrammatical)
_______________br _____bf
^*^new who house key forget
‘Why/when/how did the new who house key forget?’ (Supplementary Video 10)
(6a) Wh-question with agreement verb (grammatical)
____bf
deaf school 3-post-1 letter when
‘When did/will the deaf school post me the letter?’ (Supplementary Video 11)
(6b) Wh-question with agreement verb (ungrammatical)
____bf
^*^deaf when school 3-post-1 letter
‘The deaf when school posted/will post me a letter.’ (Supplementary Video 12)

#### 4.1.5 Relative clause sentences

Fourteen pairs of sentences consisted of a noun phrase containing a relative clause, marked with the appropriate non-manual marking, followed by a verb phrase, with seven pairs using a plain verb as in (7a) and seven using an agreement verb in the verb phrase as in (8a). No classifier constructions were used in these sentences. The ungrammatical items were produced by swapping the order of the verb phrase relative to the subject noun phrase, as in (7b) and (8b). The equivalent sentences in the ASL set included seven relative clauses using the relative pronoun that or self; as noted above, these constructions do not appear to have equivalent forms in BSL.

(7a) Relative clause sentence with plain verb (grammatical)
_____________________________br
woman cancel meeting yesterday arrive
‘The woman who cancelled the meeting yesterday has arrived.’ (Supplementary Video 13)
(7b) Example: Relative clause sentence with plain verb (ungrammatical)
___________________________br
^*^arrive woman cancel meeting yesterday
‘Arrive did the woman cancel the meeting yesterday?’ (Supplementary Video 14)
(8a) Relative clause sentence with agreement verb (grammatical)
__________________br
woman eat chocolate 3-give-1 two-pound
‘The woman who ate the chocolate gave me £2.’ (Supplementary Video 15)
(8b) Example: Relative clause sentence with agreement verb (ungrammatical)
__________________br
^*^3-give-1 two-pound woman eat chocolate
‘He/she gave/will give me two pounds did/will the woman eat the chocolate?’ (Supplementary Video 16)

#### 4.1.6 Classifier construction sentences

Fourteen pairs of sentences consisted of a noun phrase representing the ground argument followed by a noun phrase representing the figure argument and finally a verb phrase containing a classifier construction describing a motion event – specifically, an entity classifier construction, in which the hand represents a whole or part of an entity such as a person, animal or a vehicle, as in (9a). The ungrammatical items were produced by swapping the order of the verb phrase relative to the ground and figure noun phrases, as in (9b).

Boudreault and Mayberry (2006) note that classifier sentences were the most difficult to make ungrammatical for the ASL stimuli – the same was true for BSL. Boudreault and Mayberry attribute this to a lack of understanding about the use of classifier constructions compared to other structures, although it may be that constituent orders with classifier sentences are more variable than in other syntactic constructions (e.g., Johnston, Vermeerbergen, Schembri, & Leeson, 2007). The sentences containing the classifier constructions were made ungrammatical by moving the verb phrase containing the classifier construction before the ground and figure noun phrases.

(9a) Classifier construction sentence (grammatical)
bicycle boy cl:v-fall-off-cl:b
‘The boy fell off the bicycle.’ (Supplementary Video 17)
(9b) Classifier construction sentence (ungrammatical)
^*^cl:v-fall-off-cl:b bicycle boy
‘Fall off the bicycle the boy.’ (Supplementary Video 18)

### 4.2 Testing equipment and materials

The BSL stimuli were produced by a deaf native BSL signer model who was filmed with a digital video camera. During filming, the model was monitored by three fluent signers of BSL to ensure that sentences looked as natural as possible; any sentences which were thought to be problematic were refilmed until the production speed, rhythm, timing, etc. was considered acceptable. After filming and editing, video clips for the individual sentences were used in the creation of the online experiment. Stimuli were presented on a desktop computer with a 19-inch computer screen (resolution 1024 × 768) using DMDX software (Forster & Forster, 2003). This setting was used in all but two cases. For two participants, the task was run on a MacBook Pro running Windows XP; the laptop was elevated to approximate the height of the PC used to collect the data for the other participants, and two buttons on opposite sides of the keyboard marked with red and green stickers acted as the response buttons.

### 4.3 Pilot phase

Once the creation of the task was completed, a pilot session was run with three deaf native BSL signers. Participants in this pilot phase were instructed to focus on detecting errors in the structure of the stimuli, including non-manual marking, sign order and/or the use of space. All three signers responded to each of the 168 items, rejecting those that were not clear examples of either grammatical or ungrammatical sentences. The small number of rejected stimuli, together with a few stimuli in which agreement between all three native signers could not be reached, were removed. This resulted in a remaining set of 156 items. The revised task contained 120 stimuli, with the same overall balance of syntactic structures (specifically, 36 stimuli judged to be acceptable by the native signer panel were randomly selected for removal in order to maintain equal numbers of each sentence type as in the original 168 item task).

### 4.4 Participants

We recruited deaf signers with varying reported ages of acquisition of BSL: 10 native signers from deaf families who were exposed to BSL from birth and 20 non-native signers from hearing families who reported being first exposed to BSL between 2 and 18 years of age. Participants were for the most part clearly prelingually deaf (23 reported being deaf from birth; four reported becoming deaf before age 3, and three reported becoming deaf between ages 3 and 5). All participants reported severe or profound hearing loss. All participants reported that they use BSL every day as their preferred language and that they had been using BSL for 10 years or more at the time of testing. For the purposes of analysis, we separated participants into three groups: native signers (AoA from birth), early learners (AoA in early childhood between 2 and 8 years of age) and late learners (AoA after early childhood between 9 and 18 years of age), following similar age groupings from Mayberry (1993). Participants who reported their age of BSL acquisition as ‘from birth’ had at least one deaf parent whose preferred language was BSL. The early learner participants reported using BSL and/or Signed English/Sign Supported English in primary and secondary school with students outside of class, and some reported using BSL or Signed English/Sign Supported English in class as well. Primary school in the UK begins at age 5, so participants in the early learner group would have been exposed to some form of signing around that age. The late learner participants reported using Signed English/Sign Supported English and/or spoken English in class, with teachers, and outside of class in primary and secondary school; three late learners reported using BSL outside of class with other students in addition to signed/spoken English.^11^ Details about each group are shown in Table 2. Note that the higher reading ages in the late learners compared to the early learners suggest a higher level of English proficiency in the late learners, and that there may be some individuals in this group of signers with English as an L1.

Rather than determining participants’ first language based solely on self-reported age of BSL acquisition, we obtained objective measures of English proficiency via standardised reading tests. We also obtained objective measures for nonverbal IQ. By factoring out reading age and nonverbal IQ, we were able to examine the unique effect of age of BSL acquisition on BSL syntactic knowledge.

### 4.5 Procedure

Participants were tested individually by a deaf native or fluent signer of BSL (and in two cases by a hearing signer fluent in BSL); in all cases, test-specific instructions were presented in BSL on video by a deaf native signer. Informed consent was obtained from all participants. As explained above, we also administered English reading test(s) as a measure of English proficiency, in addition to the BSL Grammaticality Judgement Task. We additionally administered the nonverbal subtests of the Wechsler Abbreviated Scale of Intelligence as an independent measure of visual–spatial skills, in order to avoid non-linguistic cognitive skills as a further confound.

#### 4.5.1 Test of visual–spatial skills

Each participant was tested first on their visual–spatial skills, using the nonverbal matrix reasoning and block design subtests of the Wechsler Abbreviated Scale of Intelligence (WASI) (Wechsler, 1999). Based on the raw scores for these two subtests, *t*-scores for each subtest and an overall Performance IQ score were computed. Because subjects’ scores on the two subtests were not highly correlated (*r*(28) = .25, one-tailed *p* = .091), we kept the *t*-scores separate for analyses.

#### 4.5.2 Reading test(s)

Next, each participant was tested on their English reading skills. All participants were first given the General Reading Test-II (MacMillan Test Unit, 2000). Those who performed at ceiling on the GRT-II were additionally given the more difficult Vernon-Warden test (Vernon-Warden Reading Comprehension Test Revised, 1996). Reading age was determined based on the Vernon-Warden test if they took it; otherwise reading age was based on the GRT-II.

#### 4.5.3 BSL Grammaticality Judgement Task

The procedure was explained to the participants, followed by a practice session of eight sentences, including four grammatical sentences and their four ungrammatical counterparts. In the actual experiment, the 120 sentences were presented in four blocks of 30 sentences each and were individually randomised. There was a pause between blocks, with participants continuing the experiment whenever they felt ready. Stimuli were presented in the centre of the screen with a black frame surrounding (video dimensions 720 × 576). Participants were instructed to judge whether the sentence appeared to them to be acceptable or not by paying attention to features such as sign order and the match or mismatch of head movement and facial expressions. They were also told to respond quickly but carefully. (See Appendix A for an English translation of the full set of instructions given to participants.) Participants pressed the right button on a button-box to indicate a positive response (i.e., an acceptable sentence), and the left button to indicate a negative response (i.e., an unacceptable sentence). This mapping was changed accordingly for left-handed participants (i.e., left button for a positive response, right button for a negative response). Reaction times (RTs) were measured from stimulus onset. Each video was presented until the participants made a response, with 1000 ms after response before the next trial began.

## 5 Results

We began by examining average performance by participants and items to identify any anomalies before proceeding further. One native signer’s performance was highly atypical (accuracy more than four standard deviations lower than the mean of the other native signers) and was therefore excluded from the analyses reported here. After excluding this participant from the analysis, we averaged over subjects to determine whether any items should be excluded. We used a relatively strict criterion, excluding any item for which average performance was less than 66% correct (*p*(Accuracy > chance) > .05 by binomial test with *n *= 30). This resulted in the exclusion of 23 sentences (5 grammatical, 18 ungrammatical), leaving 97 in the set for analysis (55 grammatical, 42 ungrammatical). This reduced data set was used for all analyses reported below.

Hierarchical mixed effects regression analyses were conducted on trial-level data, fitting random intercepts for subjects and items, using restricted maximum likelihood estimation (Baayen, Davidson, & Bates, 2008). Separate models were fit for the two dependent measures: accuracy (proportion correct) and response time (considering correct responses only). For both dependent variables, we began by considering the main effects and interactions involving grammaticality (two levels: grammatical and ungrammatical), sentence type (six levels: simple, agreeing, negation, wh-question, relative clause, classifier construction), and AoA group considered categorically (three levels: native, early, and late). Subsequent analyses considered AoA as a continuous measure. Analyses also took into account video duration, as well as factors varying between participants which could affect performance on the task, including nonverbal IQ, reading age, and mean years of BSL experience (reading age and mean years of BSL experience were significantly different for the early and late signers we tested; see Table 2 and §5.1 below). For all continous predictors we first fit a model including nonlinear transformations (restricted cubic splines using three knots at quantiles .1, .5., .9), then removing all non-significant nonlinear terms from the final model. We report *p*-values estimated by Markov chain Monte Carlo simulation (*n* = 5000).

### 5.1 Accuracy

In the initial regression model (including the full factorial contrast between grammaticality, sentence type and AoA group), none of the interactions involving AoA group approached significance (*p_MCMC_* > .2). We therefore removed the categorical variable of AoA group, instead treating AoA as a continuous measure and fitting a model without interactions involving AoA. As the effects related to item characteristics appear to dissociate from effects related to participant characteristics, we will discuss them separately.

#### 5.1.1 Effects of grammaticality and sentence type

**Fig. 1:**
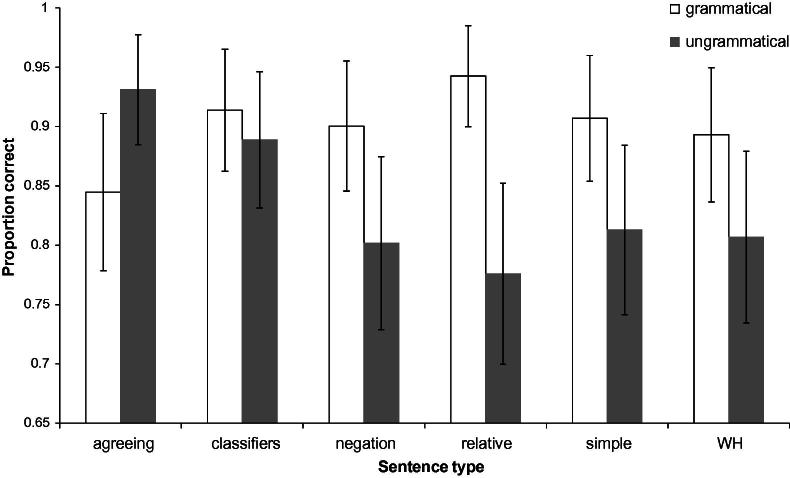
Proportion correct as a function of grammaticality and sentence type. Error bars reflect standard error of the mean by participants.

The main effect of grammaticality was significant, with an advantage for grammatical over ungrammatical sentences (95% highest posterior density interval (HPDI) of the difference = [.002, .175], *p_MCMC_* = .049). There was also an overall effect of sentence type: responses to classifier construction sentences were the most accurate (*p*(correct) = .902; significantly better than all other sentence types in pairwise comparisons, with the exception of agreeing sentences for which *p_MCMC_* = .080), and responses to negation and relative clause sentences were relatively less accurate overall (*p*(correct) = .854 and .847 respectively). These main effects were qualified by an interaction, as illustrated in Fig. 1. Simple main effect tests on grammaticality within each sentence type revealed that this interaction was largely driven by agreeing and classifier construction sentences for which the advantage for grammatical sentences was not observed (agreeing sentences: 95% HPDI of the grammatical–ungrammatical difference = [−.194, .041], *p_MCMC_* = .152; classifier construction sentences = [−.004, .221], *p_MCMC_* = .050). All other sentence types exhibited a strong advantage for grammatical sentences (*p_MCMC_* < .006). Video duration did not predict accuracy (*p_MCMC_* = .414).

#### 5.1.2 Effects of participant characteristics

**Fig. 2:**
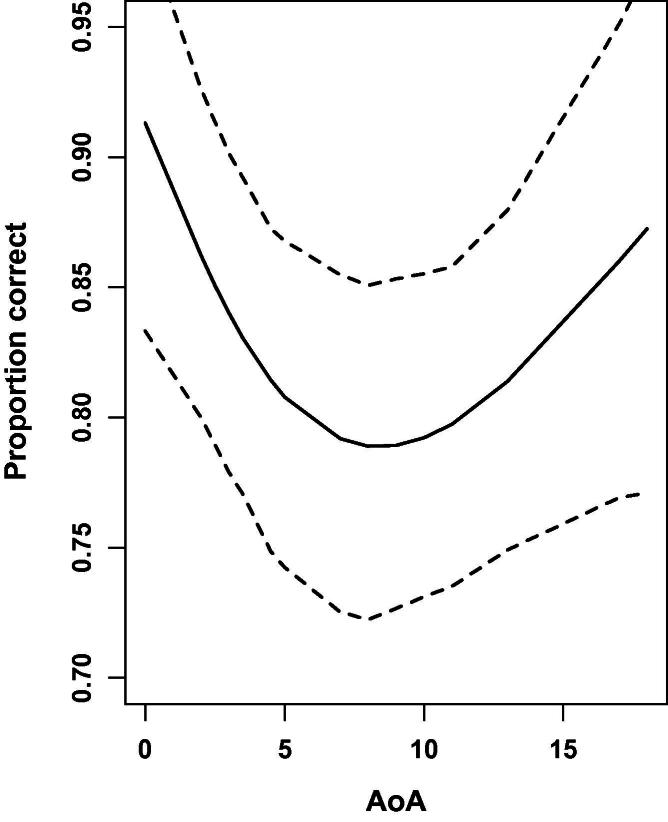
Partial effects of AoA on proportion correct. Dashed lines indicate 95% confidence interval of parameter estimates.

We investigated the partial effects of AoA, nonverbal IQ (separate measures for matrix reasoning and block design subtests), reading age, and mean years of BSL experience (i.e., identifying the extent to which variance is explained by each of these variables after controlling the others, as well as the item characteristics described in the previous section). Neither of the nonverbal IQ measures predicted accuracy (both *p_MCMC_* > .5), nor did reading age (*p_MCMC_* > .5) or years of BSL experience (*p_MCMC_* = .137). However there was a reliable effect of AoA (linear component *p_MCMC_* = .001, nonlinear component *p_MCMC_* = .004) as illustrated in Fig. 2. This effect of AoA can be characterised as high performance for native signers, with reliable linear tendency for decreasing performance with AoA until approximately age 8, beyond which change in performance as a function of AoA becomes less reliable.

In order to account for the AoA effects in early but not late learners, we additionally analysed the relationship between age of BSL acquisition and reading age. *T*-tests revealed that the mean reading age for late learners (mean = 13.4 years) was significantly higher than the mean reading age for early learners (mean = 10.7 years; *t*(*18*) = 2.01, one-tailed *p* = 0.030). Furthermore, for late learners, there was a significant zero-order correlation between English reading age and age of BSL acquisition (*r*(9) = .642, *p* = 0.031), while no such correlation was present for early learners (*r*(11) = −.139, n.s.).

### 5.2 Response times

The same steps for analyses were carried out on response time data, but considering only correct responses (87.0% of trials in the reduced data set). Response times were measured from the start of each video clip, and varying durations between items were controlled by taking video length (number of frames) into account in this analysis. We began with the same type of model as in the analysis of accuracy - that is, including the three-way interaction involving grammaticality, sentence type and AoA group, along with the other predictors: nonverbal IQ, reading age, and mean years of BSL experience. As was the case for accuracy data, there were no significant interactions involving AoA group (*p_MCMC_* > .25), so we proceeded to fit models including only the interaction between grammaticality and sentence type, and a separate continuous measure of AoA.

#### 5.2.1 Effects of grammaticality and sentence type

**Fig. 3:**
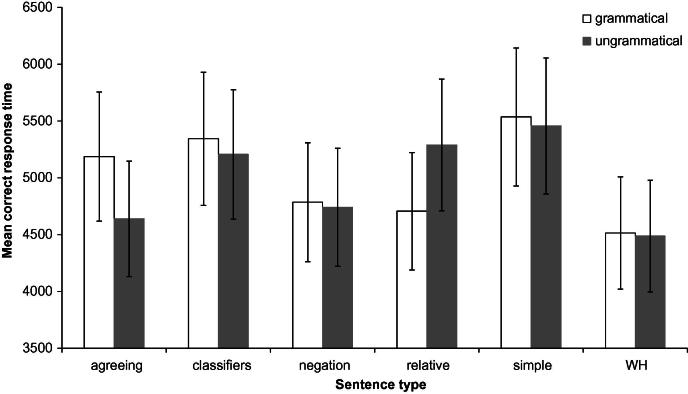
Mean correct response times as a function of grammaticality and sentence type. Error bars reflect standard error of the mean by participants.

**Fig. 4:**
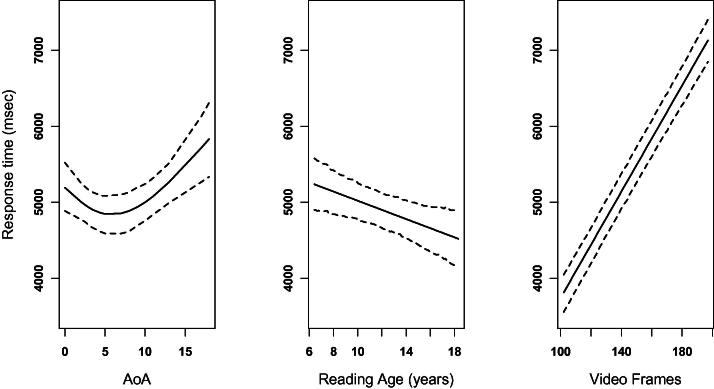
Partial effects of AoA, reading age and video duration (number of frames) on correct response times. Dashed lines indicate 95% confidence interval of parameter estimates.

The first main effect to mention is a strong and reliable effect of video duration; participants responded slower as a function of the number of frames, as shown in the right panel of Fig. 4 below (95% HPDI on the relative increase in RT per video frame = [32 ms, 37.6 ms], *p_MCMC_* < .0001), highlighting the need to take this factor into account in analysis of response times. The main effect of grammaticality on response times was not significant (95% HPDI = [−96 ms, 393 ms]), although there was a reliable effect of sentence type. Responses to wh-questions and negated sentences were reliably faster than other sentence types (mean RT for wh-questions = 4507 ms, and for negation = 4766 ms; comparisons with other sentence types yielded *p_MCMC_* < .02 with the exception of agreeing sentences which were only marginally slower than these two sentence types). Simple sentences were reliably slower than all other sentence types (*p_MCMC_* < .01), except for those involving classifier constructions (*p_MCMC_* = .21). These main effects were qualified by a significant interaction, as illustrated in Fig. 3. Tests of simple main effects revealed that there was a grammaticality effect for relative clause sentences, with grammatical sentences yielding significantly faster responses than ungrammatical sentences (95% HPDI for the difference = [203, 706], *p_MCMC_* = .002). Although no other simple effects of grammaticality reached significance in isolation (*p_MCMC_* > .3 for each of the simple main effects), agreeing sentences exhibited different performance when compared to other sentence types (e.g., *p_MCMC_* = .03 in simple 2 × 2 interaction involving only agreeing and negated sentences, with similar patterns in other simple interactions): a relative tendency for grammatical sentences to be slower than ungrammatical sentences.

#### 5.2.2 Effects of participant characteristics

Having taken item characteristics into account, we turn to investigations of how participant characteristics predict response times, looking at partial effects of factors that vary between individuals. As was the case for accuracy, neither measure of nonverbal IQ was a significant predictor (both *p_MCMC_* > .2), nor was years of BSL experience (*p_MCMC_* = .7). The partial effect of reading age was significant (linear component only); participants with a higher reading age made faster responses, as shown in the middle panel of Fig. 4 (95% HPDI for the decrease in RT per year of reading age = [14 ms, 106 ms], *p_MCMC_* = .007); although the results from analysis of accuracy demonstrate that this increased speed did not translate into more accurate performance. Crucially, the main effect of AoA also predicted response times (linear component, *p_MCMC_* = .025; nonlinear component *p_MCMC_* = .005) as illustrated in the left panel of Fig. 4. Here the effect of AoA was largely limited to later learners of BSL: AoA does not explain variation in RT among signers who learned BSL before about age 8 but does explain some of the variation in RT with later AoA beyond that point.

To summarise, regression analyses showed age of BSL acquisition to contribute unique variance in BSL syntactic knowledge in early learners when the effects of English ability (via reading) and nonverbal IQ were controlled. No such effect of age of BSL acquisition was found in late learners (after the age of 8 years). Furthermore, late learners were significantly better readers and had slower reaction times than early learners. Duration of the video stimuli predicted reaction time but not accuracy.

## 6 Discussion

In this study, native signers were more accurate than early learners in identifying grammatical and ungrammatical sentences in the BSL Grammaticality Judgement Task. Among non-native signers, regression analyses showed that early learners (those who acquired BSL between 2 and 8 years of age) showed a decrease in accuracy as age of first exposure to BSL increased, while late learners (with age of acquisition between 9 and 18 years old) did not show these effects, once measures of nonverbal IQ and English reading scores were taken into account (as illustrated in Fig. 2). Furthermore, age of BSL acquisition was significantly correlated to reading age for the late learners only. Thus, the better readers were those who learnt BSL latest. Furthermore, as a group, the late learners were better readers than the early learners (i.e., the late learners scored significantly higher on the reading tests than the early learners).^12^ Taken togther, these findings suggest that the late learners in the current study may have had first language competence in English and learned BSL as a second language. That is, they can be considered L2 signers of BSL.^13^

This study provides two important findings. Firstly, we have provided the first unequivocal evidence of L1 age of acquisition effects in a sign language. Even when factoring out the possible confounds of reading age and nonverbal IQ, we still find a significant drop in BSL grammaticality judgement as a function of age of BSL acquisition. Specifically, performance on the grammaticality judgement task (corrected for reading age and nonverbal IQ) steadily decreases until around age 8 in the early learner group. This holds true even though the syntactic violations in the ungrammatical stimuli are quite major (e.g., moving a modal into the subject or object noun phrase). This supports, and importantly strengthens, previous research which has found L1 sign language acquisition effects in signers who self-report as delayed L1 signers of a sign language (Boudreault & Mayberry, 2006; Mayberry, 1993).

Secondly, we have provided further support that delayed L1 acquisition differs from L2 acquisition of sign language. The results extend Mayberry’s (1993) findings of differences between *prelingually deaf L1* signers and *postlingually deaf L2* signers in an ASL sentence recall task. The fact that the current study has found differences in performance on the BSL Grammaticality Judgement Task in *prelingually deaf L1* signers and *prelingually deaf L2* signers has important implications.^14^ For instance, although success with literacy and spoken language amongst deaf individuals is highly variable, late learners in this study appear to have been successful in establishing English as their first language (L1). This L1 proficiency in English then may be used to scaffold learning of BSL later in life. This would explain why performance decreased as age of acquisition increased in the early learners (with AoA up to around 8 years of age) but no such effect was found in late learners who acquired BSL after that. It is important to note, however, that although the reading ages of the late learners in this study were significantly higher than those of the early learners, all groups scored substantially lower than even the minimum ‘adult’ level norms for the Vernon-Warden test, reflecting the lower level of reading skills in the deaf community (Vernon-Warden Reading Comprehension Test Revised, 1996). The fact that higher reading age across all groups correlated with faster reaction times could be a reflection of more successful experience with educational testing in school.

Another important implication of this study relates to the notion of a critical period for second language acquisition. Johnson and Newport (1989) proposed two versions of the critical period hypothesis: the *exercise* version and the *maturational state* version. The exercise version predicts that if the language learning capacity is exercised in early childhood via exposure to a first language, it will remain intact. If however, this capacity is not exercised during this time, it will disappear with maturation. On the other hand, the maturational state hypothesis states that maturation negatively affects acquisition of any language; that is, native-like acquisition of any language whether it is the first, second, third, etc. must begin early in life, since the human capacity for learning languages declines with maturation. Both versions make the same predictions about first language acquisition (i.e., that first language learners will not achieve native proficiency) but differ in their predictions about second language acquisition. The exercise version of the critical period hypothesis predicts that late first language learners will inevitably arrive at levels lower than native proficiency, while late second language learners will not necessarily do so, but may well reach fully native-like levels in their L2. The maturational state version predicts that anyone first exposed to an L2 after a critical period will only achieve non-native levels of skill. In the current study, no significant differences in BSL grammaticality judegement were found between the native signers and the prelingually deaf L2 signers, which is consistent with the exercise version of the critical period hypothesis. This is also consistent with studies which have found that high levels of proficiency can be achieved in second language learners (e.g., Birdsong, 1992; Bongaerts, 1999; Hakuta et al., 2003; White & Genesee, 1996). The slower reaction times of the late learners compared to the early learners is also consistent with other second language learning research; White and Genesee (1996) and McDonald (2000) found slower reaction times with late L2 learners than native or near-native speakers of English.

The fact that there were no significant differences between native signers and prelingually deaf L2 signers in the current study additionally means that the BSL Grammaticality Judgement Task is not suitable for use as a screening tool for BSL skill in deaf adult signers of BSL. Such a screening tool would need to show robust and strong effects across all three groups of signers.

### 6.1 Variation in performance across sentence types

Detailed analyses investigating analysis of variance amongst various factors in the BSL data revealed findings similar to those of Boudreault and Mayberry (2006). For example, overall there were more errors in judging ungrammatical sentences than grammatical sentences. This may simply reflect a general response bias against judging a sentence to be ungrammatical (however, this was not true for all sentence types as there was no such bias for grammaticality for classifier construction sentences and agreeing verb sentences, as discussed below). Also, relative clause structures were judged least accurately overall, in both languages. This is consistent with assumptions about relative clauses being learned relatively late in ASL acquisition, similarly to conditionals (Reilly et al., 1990).

Another similarity is that in both the ASL study and the current study, the classifier construction sentences were amongst the most accurately judged of all the sentence types. Based on studies which have found that classifier constructions are mastered by deaf children very late in ASL acquisition (Schick, 1987; Supalla, 1982), Boudreault and Mayberry (2006) had predicted that classifier sentences would be amongst the *least* accurately judged sentence types. The assumption made by Boudreault and Mayberry actually appears to be incorrect, because studies such as Schick (1987) and Supalla (1982) have focused primarily on the acquisition of the meaningful elements of classifier constructions themselves rather than the syntax of sentences containing classifier constructions. The classifier constructions themselves (in the current study and the ASL study) were not ungrammatical. Rather, the constituent ordering in the sentences containing the classifier constructions were made ungrammatical by changing the ordering of the figure, ground and classifier construction. The typical ordering (and the order that was used in the grammatical sentences) is ground-figure-action (i.e., noun phrase representing the ground argument followed by noun phrase representing the figure argument followed by verb phrase containing a classifier construction). The ordering that was used in the ungrammatical versions was action-figure-ground. The order ground-figure-action has also been found in gesture strings produced by hearing non-signers asked to describe motion events without speech; Gershkoff-Stowe and Goldin-Meadow (2002) use this to argue for ground-figure-action (in their terminology, stationary object-moving object-action, or SMA) as a ‘natural’ order for semantic relations. Additionally, Gershkoff-Stowe and Goldin-Meadow (2002) found that gesture strings where the action was produced first occurred very rarely. If a ground-figure-action order is a ‘natural’ or unmarked order for semantic relations to be expressed in signed languages, and an action-first order is very marked, as Gershkoff-Stowe and Goldin-Meadow argue for gesture without speech, then this could explain the relatively high accuracy of classifier sentences (both grammatical and ungrammatical) compared to the other sentence types, since the classifier sentences were made ungrammatical by moving the action to the sentence-initial position.

Other findings in the current study with sentence types also require some interpretation. For instance, for agreeing sentences only, ungrammatical sentences were judged more accurately and faster than grammatical sentences. Thus the ungrammatical agreeing sentences appear to be relatively “easy” to identify. This may be due to the particularly overt use of space in the ungrammatical agreeing sentences compared to the other sentence types. These sentences were made ungrammatical by moving the verb into the subject argument noun phrase. The model who produced the sentences usually accompanied his spatial modification of the agreeing verb with an overt turn of his torso toward the location established for the argument(s). This interruption of the subject noun phrase by a rotation in torso was quite overt and noticeable. For example , in example (4b) from §4.1.3 above, the model begins by signing sign with his torso facing the camera, then rotates his torso to the right for reject-3 then back toward the camera for class. In other ungrammatical agreeing sentences, for example those with double agreement verbs, the rotation is even more extreme. It is likely for this reason that the ungrammatical sentences were judged faster and more accurately than the other sentence types.

The opposite pattern was found with relative clause sentences. That is, for relative clause sentences, ungrammatical sentences were far less accurately judged, and judged far more slowly, than grammatical sentences. Thus in this condition, ungrammatical sentences were “difficult”. This could be explained partly by the way relative clauses are marked in BSL (via brow raise on the subject phrase and a pause before the verb phrase) and also by the way in which the relative clauses were made ungrammatical (i.e., by swapping the positions of the subject noun phrase and verb phrase). This is different from most of the other sentence types in which sentences were made ungrammatical by moving a single sign into the subject argument noun phrase. In the ungrammatical sentences, although they may be pragmatically odd, the subject phrase and the verb phrase could each be considered clauses on their own. The fact that a pause interrupts them may make it more likely for signers to try to interpret them as separate clauses. This would explain why these ungrammatical relative clause sentences were both more slowly and less accurately judged than the grammatical sentences.

### 6.2 Limitations and future work

In terms of studying critical period effects, one potential limitation of the current study is the inclusion of late learners with good English skills, who potentially had L1 proficiency in English. As noted in §3, recruitment of deaf individuals who reported learning BSL as a first language during or after late childhood was extremely difficult. If it had been possible to recruit only signers who reported learning BSL as a first language natively or in early/late childhood, this would have been one way to test L1 critical period effects more directly.

There are several ways in which the current study could be improved upon in future, particularly in an attempt to create a screening test of grammaticality judgement. One possibility would be to probe types of ungrammaticality other than those included in this study. Following Boudreault and Mayberry’s (2006) procedure for the ASL study, most of the BSL stimuli were made ungrammatical by moving a constituent into the noun phrase. This type of violation is quite major and would appear to be ungrammatical in many of the world’s languages. There are other ways of creating ungrammatical sentences – e.g., by swapping other types of constituents, or by moving functional elements such as negative or interrogative words/constituents to inappropriate positions in the sentence.

One of the challenges faced by researchers wishing to study grammaticality judgement in BSL is the extent of variability in constituent order in BSL. In fact, Deuchar (1983) and Sutton-Spence and Woll (1999) have suggested that constituent order in BSL is so variable that it is best accounted for in terms of information structure (e.g., topic/comment, given/new information), with only weakly grammaticalised constituent order constraints. The lack of understanding of variable constituent order in BSL is related to the fact that little research has been done on BSL syntax and what has been done has been based largely on small and/or unverifiable data sets. The fact that a large, representative corpus of BSL video data now exists (Schembri, Fenlon, Rentelis, & Cormier, 2011) means that studies that examine the syntactic structure in BSL, taking into account variability due to information structure, can be undertaken in the future. Once this future work is undertaken, including additional types of ungrammatical stimuli could produce results with sufficiently strong differences across groups that they could be used in a screening test, which was one of the original goals of this study.

Another potential area for improvement could be the elicitation of grammaticality judgements along a rating scale (e.g. 5-point or 7-point Likert scale) instead of a binary judgement (grammatical versus ungrammatical) (Bard, Robertson, & Sorace, 1996; Sorace & Keller, 2005). Finer grained judgements could help identify those stimulus items which are most strongly judged to be grammatical or ungrammatical. Those items with mean judgement scores at either end of the scale (e.g., in a 7-point scale, those stimuli with scores closer to 1 or 7) could therefore be most usefully included in a screening test, while those stimuli with weaker judgements, with mean scores in the middle of the scale (e.g., closer to 4) could be eliminated.

## 7 Conclusion

In summary, the current study shows significant L1 age of acquisition effects in the grammatical judgement of deaf BSL signers, when age of acquisition is between birth and around 8 years of age. No such effects were found in deaf BSL signers who acquired BSL after age 8, for whom English was likely functioning as their first language and BSL as their second. The results are consistent with Boudreault and Mayberry’s (2006) findings of L1 age of acquisition effects in prelingually deaf early learners of ASL. However, the current findings importantly strengthen Boudreault and Mayberry’s (2006) findings by showing that AoA effects are still strong even when reading age and nonverbal IQ are factored out. Thus the BSL study shows the *unique* effect of AoA on grammatical judgement in early learners. This is the first demonstration of a unique effect of AoA on signed language acquisition. This effect suggests that native signers have the strongest advantage in grammatical judgement. Amongst non-native signers who learn BSL before the age of 8 years, the earlier the acquisition of a signed language, the better the performance. Although the findings from the current study are specific to grammatical knowledge, previous studies which have found AoA effects between native signers and early L1 learners (based on self-report) suggest that this may extend to other areas such as phonology and morphology, not only in judgement but also production (e.g., Morford et al., 2008; Newport, 1990). Further research would be needed to determine if AoA contributes a unique effect in these domains.

These results are also consistent with Mayberry (1993) who found L1 age of acquisition effects in prelingually deaf early and late learners of ASL (with ASL as delayed L1) and no such effects in postlingually deaf ASL signers who had acquired spoken English naturally as an L1 before onset of hearing loss. However, the current study importantly extends Mayberry’s (1993) finding of L1 and L2 differences to prelingually deaf signers who acquired BSL late but who likely had L1 proficiency in English and therefore had acquired BSL as an L2. These late learners likely used English L1 proficiency to scaffold later BSL acquisition. This supports previous research that native-like second language acquisition is possible in some aspects of grammar (e.g., in grammaticality judgement).

Despite the fact that our results may not come from a representative sample of the adult deaf signing population (e.g., we did not include early learners who had English as an L1), it might be tempting to interpret these results as support for use of English first with deaf children. Our findings do suggest that some non-native signing deaf individuals can achieve a degree of proficiency in sign language as L2 if some proficiency in a spoken/written language such as English is achieved as L1 first, although these individuals do not do as well on the BSL Grammaticality Judgement Task as native signers. However, there are real risks in relying only on successful acquisition of English as L1 for deaf children. Research in North America has clearly shown that the success rates of both spoken language acquisition and literacy in deaf children continue to be very low (as our reading age scores can attest), even despite recent advances in amplification technology such as hearing aids and cochlear implants (Blamey, 2003; Traxler, 2000). Although recent statistics on spoken/written language acquisition in deaf children specifically in the UK are lacking (Conrad, 1979 is the most recent large scale UK study), there is no evidence to suggest the situation is different in the UK than in other countries.

One thing that seems very clear is that successful early acquisition of a first language is crucial, whether that language is a natural signed language such as BSL or a spoken/written language such as English. Relying on the acquisition of spoken language as L1 is risky among deaf children, and if it fails, successful acquisition of a signed language as L1 is unlikely as well, resulting in an overall delay in language development that many years of exposure to sign language does not appear to eliminate (Humphries et al., 2012). The current study supports many others showing that early exposure to accessible language is much more likely to result in successful language acquisition than later exposure. The advantages of early sign language exposure remain clear even with rapid advances in hearing aids and cochlear implants (Mayer & Leigh, 2010). Bilingual education is the best way of ensuring that deaf children have early exposure to both a signed language and a spoken/written language, which will provide the deaf child with the best chance for successful language acquisition, in either or both languages (Grosjean, 2001).

## Figures and Tables

**Table 1:** Participants in ASL Grammaticality Judgement Task and their % accuracy.

	*N*	AoA (mean)	AoA (range)	Mean age (range)	Mean years of ASL use (range)	Mean % accuracy on GJT (%)
Native	10	–	–	24.2 (18–41)	24.3 (18–41)	78
Early	10	5.6	5–7	43.2 (31–62)	37.6 (14–47)	68
Late	10	10.3	8–13	43.0 (24–79)	32.9 (13–71)	59

**Table 2:** Participants in BSL study.

	*N*	AoA (mean)	Mean age (range)	Mean years of BSL use (range)	Mean sum of WASI *t*-scores (range)	Mean reading age (range)
Native	10	–	39.7 (20–57)	39.7 (20–57)	113.2 (83–126, SD 14.58)	12.7 (7–16, SD 3.8)
Early	11	4.4 (2–8 years, SD 2.3)	36.5 (19–54)	32.0 (17–51)	115 (99–123, SD 10.37)	10.7 (7–17, SD 3.1)
Late	9	12.8 (9–18 years, SD 3.6)	30.9 (20–43)	18.1 (10–26)	120.9 (100–134, SD 8.1)	13.4 (10–18, SD 3.0)

## Appendix A English translation of BSL instructions given to participants for BSL Grammaticality Judgement Task

This task should take about 30 min to complete.

You will see a set of 120 sentences. Some of them will seem “right” to you. Some of them will have errors. For example, in some sentences you may think the signs are in the wrong order, and in some sentences you may feel that the facial expressions or head movements do not match the signs. After you see each sentence, if it seems “right” to you, push the green button; if it seems “wrong” to you, push the red button.

The sentences will be presented in four blocks of 30 sentences each. After each block you can take a break.

During each block, respond as quickly but as carefully as you can. Keep your hands resting on the button box at all times so you will be able to push the red or green button as fast as possible. If you are right-handed, make sure the green button is on your right; if you are left-handed, make sure the green button is on your left.

You will have a set of eight practice sentences first. If you have any questions, either before or after the practice, please ask us.

## Footnotes

3 For useful overviews covering key literature on the critical period hypothesis in second language acquisition, see Birdsong (1999a), Hyltenstam and Abrahamsson (2005) and Harley and Wang (1997).

4 With regard to syntax, Newport (1990) found no differences between native and non-native signers in the comprehension of basic word order. This was measured by a task where signers watched simple ASL sentences with three different word orders (SVO; OSV; VOS) combined with different non-manual markers to signal changes in information structure. Participants were asked to choose one of two pictures in which the subject and object were reversed. Newport (1990) used the word order task from the draft Test Battery for ASL Morphology and Syntax (Supalla et al., n.d.) which included practice items that demonstrated correct responses (Schembri et al., 2002). It is possible that the non-native signers could have learned the rule from these practice examples.

5 Mayberry and Lock (2003) claimed that the group of deaf people with a delayed first language in their study were not exposed to any accessible language at all until age 6, at which time they were enrolled in schools which used Total Communication, a bimodal strategy where signs (some from ASL, some invented for representing English grammatical morphemes) are produced in English word order. At these schools they were also taught English via lipreading, reading and writing.

6 Note that we use the term ‘agreement verb’ here for ease of comparison with the literature, but we accept the analysis proposed by Liddell (2000) that these forms do not actually mark for person agreement.

7 For a review of ‘classifier’ constructions in signed languages, and issues surrounding the terminology to refer to them, see Schembri (2003).

8 Sutton-Spence and Woll (1999) note that a negative adverbial sign not (identical in form to ASL not) is used in Scotland and in some more English-influenced varieties of BSL.

9 The fact that some of these structures are slightly different across the two languages means that studying grammaticality judgement of BSL sentences, compared to ASL sentences, helps to provide much needed cross-linguistic evidence of age of acquisition effects in sign languages.

10 Examples in (1a)–(9b) consist of a word-for-sign English gloss of each sign (in small capitals). These glosses are not the only possible English equivalents for many of these signs, but are the most appropriate translations for these sentences. Above the gloss is notation of any grammatical non-manual markers and their scope: br = brow raise (used in marking topics and relative clauses), hs = headshake (used for marking negation), and bf = brow furrow (used in marking wh-questions). Although the status of person in sign languages is controversial (see Cormier (in press) for an overview), for ease of exposition, person ‘inflections’ are marked here with numbers corresponding to the traditional notions of first, second and third persons. English translations are given underneath the glossed sentences. Because the (b) examples are all ungrammatical, English translations for these are only approximate.

11 Parental and educational details are not available for one early learner and one late learner.

12 It should be noted that the reading skills of the native signers varied considerably; this is consistent with what has been reported in the literature (e.g., Chamberlain & Mayberry, 2000; Goldin-Meadow & Mayberry, 2001). The important point here is that the native signers all had a clear L1 – BSL. Thus we focus on the reading skills of the early and late learners.

13 This is not to deny that the early learners did not have some degree of proficiency in English, since as noted above, all signers are bilingual to some degree.

14 As noted in §4.4, participants were for the most part prelingually deaf (though four participants did report becoming deaf between ages 3 and 5).
