# Supplementary figures and images for: First language acquisition differs from second language acquisition in prelingually deaf signers: Evidence from sensitivity to grammaticality judgement in British Sign Language
Source: Cognition. 2012 Jul;124(1):50–65. doi: 10.1016/j.cognition.2012.04.003 (PMC3657148; doi:10.1016/j.cognition.2012.04.003)

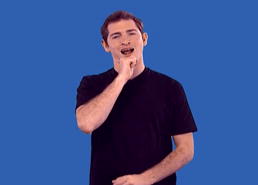

Supplement: Supplementary video 1 [file mmc1.jpg]

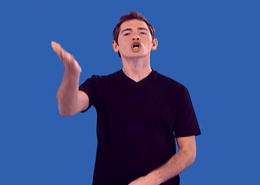

Supplement: Supplementary video 2 [file mmc2.jpg]

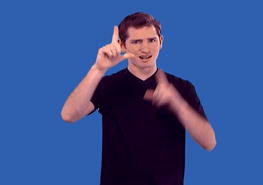

Supplement: Supplementary video 3 [file mmc3.jpg]

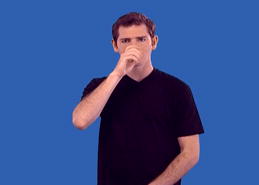

Supplement: Supplementary video 4 [file mmc4.jpg]

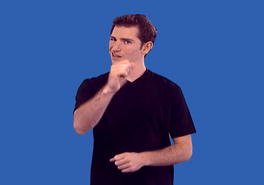

Supplement: Supplementary video 5 [file mmc5.jpg]

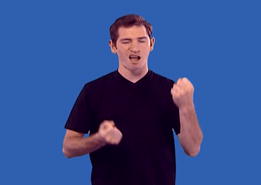

Supplement: Supplementary video 6 [file mmc6.jpg]

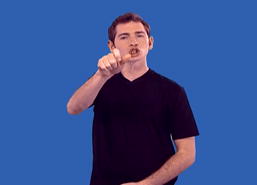

Supplement: Supplementary video 7 [file mmc7.jpg]

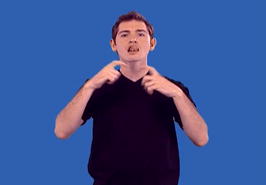

Supplement: Supplementary video 8 [file mmc8.jpg]

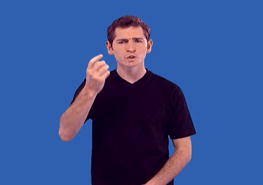

Supplement: Supplementary video 9 [file mmc9.jpg]

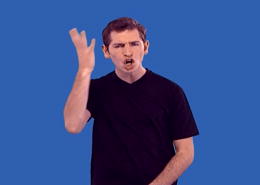

Supplement: Supplementary video 10 [file mmc10.jpg]

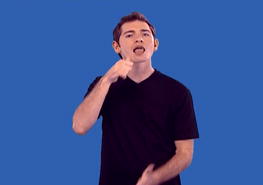

Supplement: Supplementary video 11 [file mmc11.jpg]

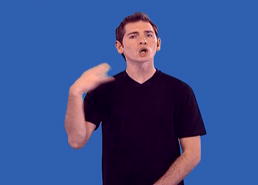

Supplement: Supplementary video 12 [file mmc12.jpg]

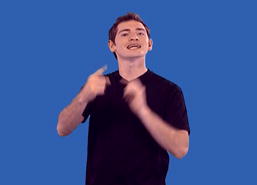

Supplement: Supplementary video 13 [file mmc13.jpg]

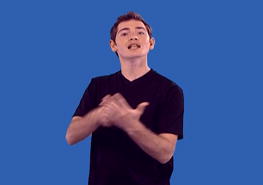

Supplement: Supplementary video 14 [file mmc14.jpg]

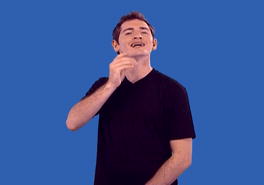

Supplement: Supplementary video 16 [file mmc16.jpg]

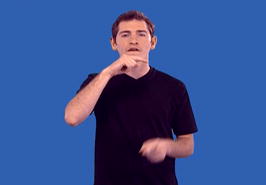

Supplement: Supplementary video 17 [file mmc17.jpg]

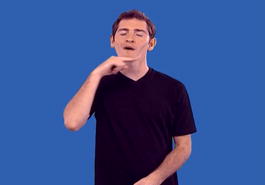

Supplement: Supplementary video 18 [file mmc18.jpg]
